# Maternal Cigarette Smoking during Pregnancy and Offspring Externalizing Behavioral Problems: A Propensity Score Matching Analysis

**DOI:** 10.3390/ijerph7010146

**Published:** 2010-01-14

**Authors:** Brian B Boutwell, Kevin M Beaver

**Affiliations:** 1 College of Criminal Justice, Sam Houston State University, Huntsville, TX 77341-2296, USA; E-Mail: bbb03@fsu.edu; 2 College of Criminology and Criminal Justice, Florida State University, Tallahassee, FL 32306-1127, USA

**Keywords:** behavioral problems, cigarette, childhood, prenatal, smoking

## Abstract

A body of empirical research has revealed that prenatal exposure to tobacco smoke is related to a host of negative outcomes, including reduced cognitive abilities, later-life health problems, and childhood behavioral problems. While these findings are often interpreted as evidence of the causal role that prenatal exposure to tobacco smoke has on human phenotypes, emerging evidence has suggested that the association between prenatal exposure to tobacco smoke and behavioral phenotypes may be spurious. The current analysis of data from the Early Childhood Longitudinal Study, Birth Cohort (ECLS-B) revealed that the association between prenatal exposure to cigarette smoke and externalizing behavioral problems was fully accounted for by confounding factors. The implications that these findings have for policy and research are discussed.

## 1. Introduction

A long line of empirical research has revealed that antisocial conduct, including early childhood behavioral problems, is heavily influenced by genetic factors. Results culled from behavioral genetic research, for example, have consistently indicated that at least 50 percent of the variance in antisocial phenotypes is attributable to genetic influences, with environmental factors explaining the remaining variance [1,2]. More specifically, the environments that have been identified as being the most salient to human development are nonshared environments, which are environments that are unique to each sibling [3,4]. Shared environments, or environmental factors that are equally experienced by all siblings from the same household, tend to have very little, if any, effect on behavioral phenotypes [5–8]. The differential effects of shared environments *versus* nonshared environments have been highlighted in the behavioral genetic literature. To illustrate, the results of four meta-analyses have converged to show that shared environments explain between 0–10 percent of the variance in antisocial phenotypes, while nonshared environments account for about 40 percent of the variance [9–12].

Given that the nonshared environment explains such a large percentage of the variance in behavioral phenotypes, there has been great interest in trying to identify the specific nonshared environments that are related to antisocial behaviors. Much of this research has focused on differential parental treatment, differential exposure to antisocial peer groups, and differential stochastic experiences [1,13–15]. But, perhaps one of the most salient nonshared environments—and one that has also been the source of a considerable amount of research attention—is the prenatal environment [16]. Compromised prenatal environments have been linked with a wide array of maladaptive outcomes, including reduced cognitive abilities, mental retardation, mental illnesses, and even antisocial behaviors [17,18].

One of the main ways in which prenatal environments are thought to affect later-life behavioral problems is through exposure to toxins, such as teratogens and neuroteratogens [16,19]. Exposure to cigarette smoke *in utero* represents what is perhaps the most widely researched toxin as it relates to childhood conduct problems [20]. Prenatal exposure to some of the agents found in cigarette smoke, especially nicotine, has been found to disrupt normal fetal brain growth, to be linked to a smaller head circumference, to produce learning and memory impairments, and to generally reduce executive control processes ([19], for a general overview see [21]). These findings are particularly important because there is a solid knowledge base linking neuropsychological deficits and executive dysfunctions to antisocial behaviors, including externalizing problem behaviors in childhood [17,22,23]. As a result, the effect that prenatal exposure to cigarette smoke has on behavioral problems may be mediated by neuropsychological impairments.

A considerable amount of research has examined the connection between prenatal exposure to cigarette smoke and behavioral problems in childhood, delinquency in adolescence, and crime in adulthood. In general, the results of at least 40 studies have revealed that exposure to cigarette smoke in utero is a risk factor for neurological dysfunction, as well as antisocial behaviors across all stages of the life course [22,24]. For example, Wakschlag and her colleagues [25] recently examined a nationally representative sample of respondents in order to explore the effects that prenatal exposure to cigarette smoke had on a range of antisocial outcomes. The study revealed that individuals exposed to cigarette smoke in utero were more likely to exhibit symptoms of oppositional defiant disorder and to have an early onset of delinquency.

The available evidence indicates a significant and positive association between prenatal exposure to cigarette smoke and later-life behavioral problems, leading some scholars to believe that this association is causal [25,26]. However, the methodologies that have been employed to estimate the effect of prenatal exposure to cigarette smoke on antisocial phenotypes are limited in their ability to rule out other rival explanations. Most notably, the association between prenatal exposure to cigarette smoke and behavioral problems later in life may be spurious owing to confounding. Ultimately, there are at least two reasons why this may be the case [27].

First, there are certain maternal characteristics that are correlated with both childhood behavioral problems and prenatal cigarette smoking. Specifically, maternal smoking is most common among women with antisocial traits [27]. Evidence is also accumulating that suggests the effects of prenatal smoking may cease to exert a significant effect on childhood behavioral problems after controlling for maternal antisocial behavior. Silberg and her colleagues [28] tested this possibility by analyzing a sample of over 500 male twins drawn from the Virginia Twin Study of Adolescent Behavioral Development. The findings of the study revealed that after controlling for familial factors, including maternal antisocial characteristics, prenatal smoking was no longer significantly associated with antisocial behavior in children.

The second reason that the relationship between maternal smoking and behavioral problems may be confounded is because of genetic factors. Specifically, researchers have suggested that maternal cigarette smoking during pregnancy might signify an underlying genetic risk for antisocial behavior [26,27,29]. A wealth of studies, examining thousands of twin pairs, has revealed that there is a sizeable genetic influence on the development of antisocial traits [10,12]. As a result, mothers who smoke while pregnant may be passing along the genetic propensity for antisocial phenotypes to their offspring. If this is the case, then the association between prenatal exposure to cigarette smoke and offspring behavioral problems may be the result of genetic transmission, not exposure to cigarette smoke in utero. Maughan and her colleagues [27] examined this possibility by employing a sample of twins drawn from the Environmental Risk (E-Risk) Longitudinal Twin Study. The results revealed the presence a significant dose-response effect of prenatal smoking on behavioral problems. Most notably, however, the researchers found that over half of the association between maternal smoking and behavioral problems was due to the influence of shared genetic factors. In other words, when a number of other risk factors were taken into account—including, maternal psychopathology, family disadvantage, and genetic effects—the strength of the relationship between maternal smoking and behavioral problems was either markedly reduced or completely eliminated. This study provides some initial evidence that the association between maternal smoking during pregnancy and offspring behavioral problems might be largely or partially driven by genetic confounds [but see 20].

Taken together, the available evidence suggests that the association between prenatal exposure to cigarette smoke and subsequent behavioral problems is complex and may be the result of confounding factors. However, much more research is needed that specifically addresses the issue of confounding before any definitive conclusions can be drawn. The current research seeks to provide a cautious step in this direction by examining the influence of prenatal exposure to cigarette smoke on externalizing behavioral problems in children by using propensity score matching (PSM) to help correct for confounding.

## 2. Methods

### 2.1. Study Population

The current research uses data from the Early Childhood Longitudinal Study, Birth Cohort (ECLS-B). The ECLS-B is a multi-wave, nationally representative study of children born in the United States in 2001. A more detailed description regarding the sample has been published elsewhere [30]. Briefly, three waves of data have been collected and are currently available to researchers. Each wave of data included a range of measures for mothers, fathers, and their biological children. Data collection began when the focal child was 9 months old and continued until they were preparing to enter kindergarten (around the age of 4 years old). The total sample size was approximately N = 10,600.

Wave I interviews were conducted between the fall of 2001 and the fall of 2002 when mothers and fathers were interviewed via telephone surveys about their child’s health, their home environment, and their overall development and wellbeing. Wave II surveys were completed between the fall of 2003 and the fall of 2004 when the children were two years old. Parental questionnaires during Wave II included items regarding socioeconomic status, physical and emotional well-being, educational achievements, and the quality of their relationship with their spouse/romantic partner.

The third wave of the ECLS-B occurred as the child was preparing to enter kindergarten. At this point, the parental surveys were altered in order to include more age-appropriate items for their children. During Wave III interviews, a number of the focal children had also been placed with professional daycare providers and so the final wave of the ECLS-B included an interview with the child’s daycare provider. After the removal of missing data, the sample size for the current study ranged between N = 3,343 and N = 3,402 prior to forming matched groups.

## 3. Measures

### 3.1. Dependent Variable

The ECLS-B contained a number of items intended to assess behavioral, cognitive, and emotional development in preschool aged children. Measures from the Preschool and Kindergarten Behavior Scales–Second Edition (PKBS-2) were included during Wave III interviews with the child’s primary caregiver. The full length version of the PKBS-2 contains 42 items designed to tap problem behaviors and social adjustment in children [31]. A number of studies have examined the PKBS-2 and found it to be a reliable and valid method of measuring disruptive behavior early in the life-course [31–33]. An abbreviated version of the PKBS-2 containing 24 of the original 42 questions was available in the ECLS-B.

Principal components analysis of all 24 measures revealed that eight items loaded on a single construct which captured variation in aggressive and disruptive behavior. Reliability coefficients were examined and the results revealed a moderate level of internal consistency among the eight measures (α = 0.74). Additional analysis revealed that removing any of the items would not significantly improve the observed alpha levels. The items used to construct the scale asked the primary caregivers (usually the mothers) how often their child was physically aggressive, easily angered, impulsive, overly active, and prone to tantrums. Parents were also asked about their child’s ability to concentrate, how often they annoyed other children, and how often they destroyed their possessions. Responses to each item were coded such that higher scores reflected increased levels of behavioral problems (*i.e.*, 1 = never, 2 = rarely, 3 = sometimes, 4 = often, and 5 = very often). The items were summed to create the childhood externalizing behavioral problems scale.

### 3.2. Treatment Variables

#### 3.2.1. Maternal Smoking

In order to assess prenatal exposure to cigarette smoke, we relied on a measure of maternal smoking obtained from the children’s birth certificates. The use of birth certificates to measure prenatal smoking has been employed by a number of researchers [34,35]. Despite some debate regarding the reliability of self-reported maternal smoking measures drawn from birth certificates [36], studies have suggested that this type of measure is reliable and not likely to bias the results of empirical research [37,38]. For the current study, maternal smoking was coded dichotomously where 0 = non-smoker and 1 = smoker. Overall, the birth certificates of the children in the sample revealed that 354 of the mothers—approximately 10 percent of the final analytical sample—smoked while they were pregnant.

#### 3.2.2. Heavy Maternal Smoking

The birth certificates of each child also included the average number of cigarettes smoked per day by the mother while she was pregnant, with responses ranging from 0 to 60 cigarettes. Following the lead of previous researchers [39], we recoded this item into a dichotomous measure of heavy maternal smoking where 0 = smoked less than 10 cigarettes per day and 1 = smoked 10 or more cigarettes per day. Overall, 174 mothers—or about 5 percent of the final analytical sample—were heavy smokers during their pregnancy.

### 3.3. Maternal Covariates

#### 3.3.1. Maternal Antisocial Behavior

Antisocial mothers are at-risk for raising disruptive and aggressive children [40]. Additionally, mothers who exhibit antisocial traits also tend to exhibit an increased propensity to smoke during their pregnancy [27]. As a result, maternal antisocial behavior may confound the relationship between prenatal exposure to cigarette smoke and behavioral problems. During Wave I interviews, mothers were administered a series of four questions intended to gauge their levels of antisocial behavior. Specifically, the participants were asked if they had ever been suspended or expelled from school, if they had ever spent the night in a mental facility, if they had ever been fired from their places of employment, and if they had ever been arrested (0 = no and 1 = yes). The items were summed to create a maternal antisocial behavior index.

#### 3.3.2. Maternal Substance Abuse

Researchers have suggested that mothers prone to substance abuse are also those with a higher predisposition to smoke while pregnant [26,27]. In other words, children exposed to prenatal smoking are also at risk of exposure to additional deleterious substances including alcohol and other drugs [20,22]. Furthermore, parental substance abuse has been tied to the development of behavioral problems in children [27]. To take account of these findings—and to avoid problems with confounding—we included a measure of maternal substance abuse drawn from Wave I of the ECLS-B. To assess substance abuse behaviors, mothers were asked four questions related to their levels of nicotine and alcohol consumption. For example, mothers were asked whether or not they had ever been convicted of driving under the influence (*i.e.*, a DUI conviction), whether they had smoked at least 100 cigarettes in the past, and whether they had ever been diagnosed with a drinking or drug problem (0 = no and 1 = yes). The individual items were summed to create a maternal substance abuse index.

#### 3.3.3. Maternal Depression

Maternal depression has been linked with an array of adverse developmental outcomes in children. Specifically, the children of depressed mothers are more likely to exhibit disruptive behavioral problems [41]. Prior research has also suggested that maternal depression may partially account for the link between prenatal smoke exposure and behavioral problems [25,27]. To account for these findings, we included a measure of maternal depression in the analyses. In order to assess depressive symptoms in mothers, the ECLS-B included a modified version of the Center for Epidemiologic Studies Depression Scale (CES-D) [42]. The CES-D has been used by researchers to distinguish symptoms of depression in both clinical and general population samples, and a number of studies have found the CES-D to be a reliable and valid measure of depression across age groups and gender categories [42–44]. During Wave I interviews, mothers responded to 12 self-administered items designed to tap instances of depression. Factor analysis revealed that each component loaded significantly on one latent construct, and calculations of reliability estimates revealed a substantial degree of internal consistency among the items composing the depression scale (α = 0.87).

#### 3.3.4. Maternal Educational Attainment

Low maternal educational attainment is correlated with the propensity to smoke during pregnancy and so failing to control for educational achievement could yield biased results [27]. As a result, an educational attainment measure was included in the analyses. During Wave I interviews, mothers were asked to indicate the highest level of education that they had presently achieved. Responses ranged from 1 = 8^th^ grade or below to 9 = Doctorate/Professional degree.

#### 3.3.5. Maternal Age

The children of very young mothers are at an increased risk of developing severe behavioral problems [45–47]. Additionally, young motherhood has been linked with an increased propensity to smoke while pregnant, and research has revealed that maternal age should be included in studies of prenatal smoking in order to avoid the possibility of confounding [26]. Maternal age was included in the analyses and measured continuously (in years).

#### 3.3.6. Family Adversity

Adverse rearing environments have been linked with chronic behavioral problems in children [17]. To account for the effects of disruptive home environments, we included a measure of family adversity in the statistical models. During Wave I interviews, mothers were asked 10 questions related to how often in the past month they argued with their spouse regarding children, financial concerns, and alcohol abuse. Responses were coded such that 1 = never, 2 = hardly ever, 3 = sometimes, and 4 = often. The items were then summed together to create the family adversity scale, where higher values indicate more adversity (α = 0.80).

#### 3.3.7. Delivery Intervention Index

Research has revealed that a number of delivery interventions are risk factors for later developmental disorders [48]. For example, psychopathology in childhood has been linked with several birthing interventions, including cesarean section deliveries, the use of forceps during delivery, and breech births [41]. To account for these findings, we included an index of delivery methods used during the child’s birth. The children’s birth certificates included information related to five interventions, such as the use of forceps, cesarean delivery, repeat cesarean delivery, vaginal delivery following a cesarean section, and the use of a vacuum during delivery. The items were coded dichotomously, such that 0 = method not used and 1 = method used. All of the measures were summed to create a delivery interventions index.

#### 3.3.8. Labor Complications Index

Labor complications during birth may increase the risk of neuropsychological impairments in children, leading to antisocial behavior [17]. Moreover, correlations between labor complications and behavioral problems could confound the relationship between prenatal exposure to cigarette smoke and externalizing behavioral problems. As a result, we included a measure of labor complications in the current analysis. The current study relied on 16 measures of labor complications drawn from the birth certificates of the children in the sample. Each item was coded dichotomously where 0 = complication not reported and 1 = complication reported. All of the items were added together to create a labor complications index.

#### 3.3.9. Apgar Scores

Apgar scores are used to capture the overall health and wellbeing of infants shortly after birth. Low Apgar scores have been tied to delayed neurological development, and previous research has found an association between Apgar scores and antisocial behavior later in the life-course [49]. If left unmeasured, Apgar scores may artificially inflate the association between maternal cigarette smoking and childhood behavioral problems. All of the analyses included the five-minute Apgar score which was drawn directly from the child’s birth certificate.

### 3.4. Paternal Covariates

#### 3.4.1 Paternal Antisocial Behavior

Research has revealed that the children of antisocial fathers are at-risk for exhibiting disruptive behavioral problems early in the life course [50]. As a result, we included a measure of paternal antisocial behavior collected at Wave I. The individual measures were identical to those asked to the mother during the same wave. For example, fathers were asked if they had ever been arrested, whether they had ever spent the night in a mental facility, whether they had ever been fired from their place of employment, and whether they had ever been expelled or suspended from school. Each of the four items was coded dichotomously (0 = no, 1 = yes) and summed together to create a paternal antisocial behavior index.

#### 3.4.2. Paternal Substance Abuse

Antisocial fathers have an increased risk of raising antisocial children and one explanation for this link is that the children of antisocial fathers are more likely to be exposed to adverse rearing environments—including those marked by paternal substance abuse [51]. To take this possibility into account, we included a measure of paternal substance abuse in the current study. During Wave I interviews, fathers were asked a series of four questions intended to assess their levels of cigarette smoking and alcohol consumption. For example, fathers were asked whether they currently smoked cigarettes, whether they had ever been convicted of driving under the influence, and whether they had ever been diagnosed with a drinking or drug problem (0 = no and 1 = yes). All of these items were summed to create the paternal substance abuse index.

#### 3.4.3. Paternal Depression

In order to measure paternal depression we relied on the same modified version of the Center for Epidemiologic Studies Depression Scale (CES-D) scale that was administered to the mothers during Wave I [42]. As noted previously, the CES-D has been validated in general populations, as well as with individuals who have been clinically diagnosed with depression [43,44]. During Wave I interviews, fathers responded to 12 items designed to assess levels of depression (α = 0.87). Higher scores reflect increased levels of depressive symptoms.

### 3.5. Demographic Characteristics

The child’s race (0 = non-white, 1 = white) and sex (1 = male, 2 = female) were drawn from Wave I of the ECLS-B. We included both variables as covariates in the study in order to avoid confounding due to excluded demographic variables.

## 4. Plan of Analysis

To examine the effects of prenatal smoking on childhood behavioral problems we employ propensity score matching analysis (PSM). PSM is a quasi-experimental design that has been used across disciplines in order to isolate treatment effects on a number of outcomes using observational data [52–54]. Matching based on propensity scores can be used in situations where a true experimental design is not feasible. For example, it would be both unethical and impractical to randomly assign mothers to smoking and non-smoking groups during their pregnancy.

The PSM analysis for the current study proceeded in a series of interrelated steps. First, we examined whether prenatal exposure to cigarettes was associated with increased externalizing behavioral problems by using the dichotomous treatment measure of maternal smoking (0 = non-smoker, 1 = smoker). Second, we examined whether the relationship between smoking and behavioral problems would remain after accounting for confounding among the variables by using PSM. To address this issue, propensity scores were estimated for the mothers in the study based on the fourteen covariates. Multivariate logistic regression models were used to generate log odds which were converted to conditional probabilities ranging in value from 0 to 1 (*i.e.*, the probability that a mother smoked during her pregnancy). Subjects were matched based on these probabilities using 3-to-1 nearest neighbor matching with a matching caliper of 0.05. Third, and finally, we repeated the steps outlined above using the treatment variable of heavy maternal smoking (0 = mother smoked less than 10 cigarettes, including non-smokers, 1 = mother smoked 10 or more cigarettes per day).

## 5. Results

The results of the pre- and post-matching independent samples *t*-tests for the fourteen covariates are presented in Table 1. Prior to matching on propensity scores, the subjects in the sample differed on eleven out of the fourteen covariates. The differences between subjects offer some initial evidence that the relationship between prenatal nicotine exposure and behavioral problems may be confounded (*i.e.*, influenced by unmeasured variables). After matching based on propensity scores, we were able to eliminate significant differences between the treatment and control groups in the sample (*i.e.*, the children of mothers who smoked during their pregnancy, and those who did not).

**Figure 1.**
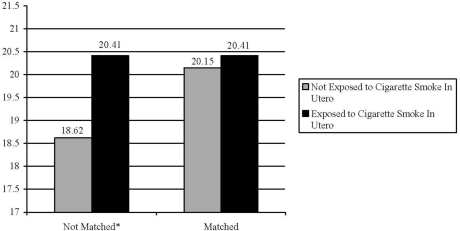
The association between Exposure to Prenatal cigarette smoke and Externalizing problem behaviors in children. *P ≤ 0.05; Notes: Unmatched Sample (n = 3,402), Matched Sample (n = 1,064).

Once statistically equivalent groups were established, we were able to directly examine the effect of any exposure to prenatal smoking on externalizing behavioral problems in children. Figure 1 displays the effects of maternal smoking on externalizing behaviors both before and after matching.

Prior to being placed into matched groups, the children of mothers who smoked during their pregnancy exhibited higher levels of externalizing problem behaviors when compared to the children of non-smoking mothers (mean difference = 1.80, *t-*value = 6.98, *P* ≤ 0.05). Once subjects were placed into matched groups, prenatal exposure to cigarette smoke was no longer a significant predictor externalizing behavioral problems (mean difference = 0.30, *t-*value = 0.74, *P* > 0.05)

The results of the balancing estimates for the treatment indicator of heavy maternal smoking are presented in Table 2. This analysis revealed that prior to matching, the participants differed significantly on ten out of the fourteen covariates in the study. After respondents were matched on propensity scores, none of the covariates differed significantly between the treatment and control groups.

**Figure 2.**
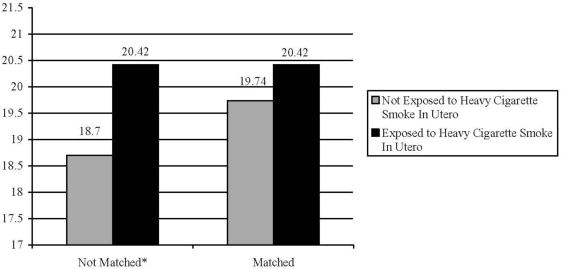
The association between heavy exposure to prenatal cigarette smoke and externalizing problem behaviors in children. *P ≤ 0.05; Notes: Unmatched Sample (n = 3,343), Matched Sample (n = 522).

The association between exposure to high levels of maternal smoking and externalizing behavioral problems is presented in Figure 2. Prior to matching, the children exposed to high levels of cigarette smoke in utero also had significantly higher scores on the externalizing problem behaviors scale when compared to those children not exposed (mean difference = 1.72, *t-*value = 4.83, *P* ≤ 0.05). After matching on the fourteen covariates, the effect of heavy maternal smoking on externalizing behavioral problems was no longer statistically significant (mean difference = 0.70, *t-*value = 1.36, *P* > 0.05).

## 6. Discussion

Researchers in the medical and behavioral sciences have long been concerned with the deleterious effects of prenatal smoking on offspring development. Early research on the topic demonstrated links between prenatal smoking and low birth weight, respiratory dysfunction, delayed motor development, and a host of other adverse health outcomes in children [19]. The consistent and robust body of findings linking maternal smoking during pregnancy with offspring’s health in early childhood prompted numerous warnings to expectant mothers cautioning them against the harmful effects of prenatal nicotine use on a developing fetus.

Subsequent research has demonstrated a statistically significant and consistent association between exposure to cigarette smoke in utero and behavioral problems over the life course. The bulk of this research, however, has failed to adequately address the methodological issues that may render the association between the prenatal smoking and externalizing problems spurious. We addressed this shortcoming in the literature by estimating the effect of prenatal exposure to cigarette smoke on childhood externalizing behavioral problems by employing propensity score matching (PSM). Consistent with the extant literature, our analysis of the ECLS-B data revealed a significant and positive association between maternal smoking and externalizing behavioral problems prior to directly modeling confounding factors. Once confounding was taken into account by estimating PSM models, the effect of prenatal exposure to cigarette smoke on childhood behavioral problems was no longer statistically significant. This null effect was observed for any exposure to cigarette smoke in utero as well as high levels of cigarette smoke exposure in utero.

The results of the current study stand in stark contrast to research revealing a direct effect of prenatal exposure to cigarette smoke on behavioral problems, leading to the question of what accounts for these disparate results. The main aspect that distinguishes the current study from most prior research is that we directly addressed confounding by using PSM techniques. Had we not modeled confounding directly, the results of our study would have been in line with those generated in previous research. We should add, however, that our findings dovetail with the results of some of the more methodologically rigorous studies that control for both genetic and environmental confounds [20,25,26]. These studies have either failed to detect any association between prenatal exposure to cigarette smoke and behavioral problems later in life or found very attenuated associations. Interestingly, research using animal models, where randomization is possible, has consistently revealed a link between prenatal exposure to tobacco smoke and subsequent impairments [22,23,25]. Perhaps the reason for these differential findings is tied to the outcome of interest—in the current study, the outcome of interest was externalizing behavioral problems early in life. More research is needed on this topic before any definitive conclusions can be drawn about the factors that might be accounting for the different findings that are generated from animal models and from those generated from human models using PSM.

## 7. Conclusion

Taken together, the results of our study, along with those that have adequately accounted for confounding [20,25,26,55], tend to call into question whether prenatal exposure to cigarette smoke has a significant *main* effect on behavioral problems. The null effect of prenatal exposure to cigarette smoke necessarily raises ethical questions about whether our study is advocating for women to continue to smoke throughout their pregnancy. There are at least four reasons why such a position is unfounded. First, replication studies that analyze other samples are needed before the results of the current study should be accepted. And, even if the results are replicated and prenatal exposure to cigarette smoke does not have an effect on behavioral problems, exposure to cigarette smoke in utero has been linked to various other outcomes, such as cognitive abilities [16]. As a result, reducing smoking among pregnant women should increase a range of prosocial and adaptive outcomes that cut across various domains of healthy human development.

Second, the current study only examined the main effect that prenatal exposure to cigarette smoke had on childhood behavioral problems. Existing research has indicated that exposure to cigarette smoke may have its strongest effects on antisocial phenotypes when it is paired with certain environments [18]. In this way, the effect of prenatal exposure to cigarette smoke is likely to be dependent on the presence of certain environmental factors. Third, and relatedly, there is some evidence to indicate certain genotypes may exacerbate or blunt the negative effects of prenatal exposure to cigarette smoke. For example, Hong *et al.* [56] found that polymorphisms in the GSTM1 and GSTT1 genes moderated the effect of prenatal exposure to cigarette smoke on birth weight. Whether other genetic polymorphisms might moderate the effect of prenatal exposure to cigarette smoke on antisocial behaviors remains an open-empirical question awaiting future research.

The fourth reason to use caution in interpreting the results of the current study is because we only examined behavioral problems measured in childhood. Perhaps the effects of prenatal exposure to cigarette smoke vary across different sections of the life course, with the effects becoming more intense over time. We do not know of any research testing this possibility, but future researchers should explore the potential age-graded nature of the effects of prenatal exposure to cigarette smoke on behavioral problems in childhood, adolescence, and adulthood.

Public policies aimed at reducing prenatal smoking draw heavily on research that has linked exposure to cigarette smoke in utero with myriad physiological, developmental, and cognitive delays in children. Our findings do not obviate the importance of these smoking prevention efforts. However, our results do suggest that the association between maternal smoking during pregnancy and childhood externalizing behavioral problems is highly complex, perhaps more complex than originally thought. As a result, much more research is needed to identify the various ways in which prenatal exposure to cigarette smoke may interfere with normal human development.

## Figures and Tables

**Table 1.** Achieving statistical equivalence between smokers and non-smokers: pre- and post-matching *t*-tests.

	**Unmatched Sample**			**Matched Sample**		
	Smoker	Non-Smoker	t-value	Smoker	Non-Smoker	t-value
***Maternal Covariates***						
Antisocial Behavior	0.77	0.20	18.40*	0.77	0.70	1.31
Substance Abuse	1.74	0.10	18.91*	1.74	1.76	−0.22
Depression	18.20	16.30	7.21*	18.20	18.10	0.24
Education	4.04	5.70	−16.47*	4.04	3.94	0.97
Age	26.60	29.10	−7.61*	26.60	26.70	−0.21
Family Adversity	19.82	18.70	4.40*	19.82	19.51	0.83
Apgar Scores	8.80	8.80	−0.47	8.80	8.70	0.87
Delivery Interventions	0.50	0.42	1.31	0.50	0.50	−0.45
Labor Complications	0.70	0.51	3.32*	0.70	0.61	0.86
***Paternal Covariates***						
Antisocial Behavior	1.13	0.52	12.74*	1.13	1.15	−0.27
Substance Abuse	3.40	2.65	7.38*	3.40	3.30	0.42
Depression	16.64	15.43	4.90*	16.64	16.24	1.06
***Demographic Covariates***						
Child’s Sex	1.50	1.50	−1.26	1.50	1.50	−0.15
Child’s Race	0.72	0.63	3.38*	0.72	0.74	−0.42

* P ≤ 0.05.

**Table 2.** Achieving statistical equivalence between heavy and light-smokers: pre- and post-matching *t*-tests.

	**Unmatched Sample**			**Matched Sample**		
	Smoker	Non-Smoker	t-value	Smoker	Non-Smoker	t-value
***Maternal Covariates***						
Antisocial Behavior	0.82	0.22	13.79*	0.82	0.80	0.60
Substance Abuse	1.70	0.14	12.54*	1.70	1.53	1.02
Depression	18.50	16.35	5.77*	18.50	18.31	0.27
Education	3.84	5.64	−12.63*	3.84	3.80	0.50
Age	26.90	29.00	−4.66*	26.90	26.60	0.41
Family Adversity	19.60	18.71	2.42*	19.60	19.61	−0.03
Apgar Scores	8.80	8.80	−0.44	8.80	8.71	0.40
Delivery Complications	0.41	0.42	−0.22	0.41	0.41	0.04
Labor Complications	0.63	0.52	1.85	0.63	0.60	0.50
***Paternal Covariates***						
Antisocial Behavior	1.13	0.55	8.75*	1.13	1.14	−0.10
Substance Abuse	3.30	2.70	4.60*	3.30	3.24	0.25
Depression	16.41	15.50	2.70*	16.41	16.20	0.46
***Demographic Covariates***						
Child’s Sex	1.50	1.50	0.23	1.50	1.50	0.36
Child’s Race	0.80	0.63	3.23*	0.80	0.74	0.20

* P ≤ .05
